# Supplementary figures and images for: Surface exclusion of IncC conjugative plasmids and their relatives
Source: PLoS Genet. 2024 Oct 9;20(10):e1011442. doi: 10.1371/journal.pgen.1011442 (PMC11493245; doi:10.1371/journal.pgen.1011442)

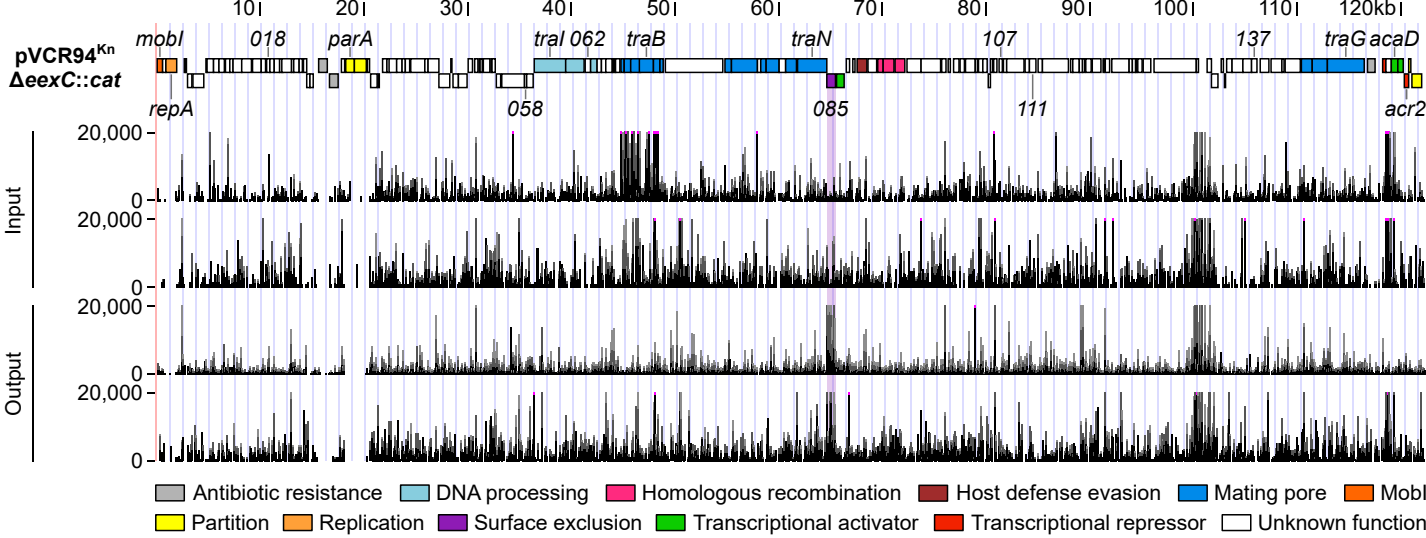

Supplement: S1 Fig — The tracks plot the number of reads from two independent replicates as a function of position in pVCR94Kn ΔeexC::cat for both the input and output libraries. ORFs with similar functions are colour-coded as indicated in the figure. This figure was created using the UCSC Genome Browser (http://genome.ucsc.edu) [89]. (PDF) [file pgen.1011442.s001.pdf]

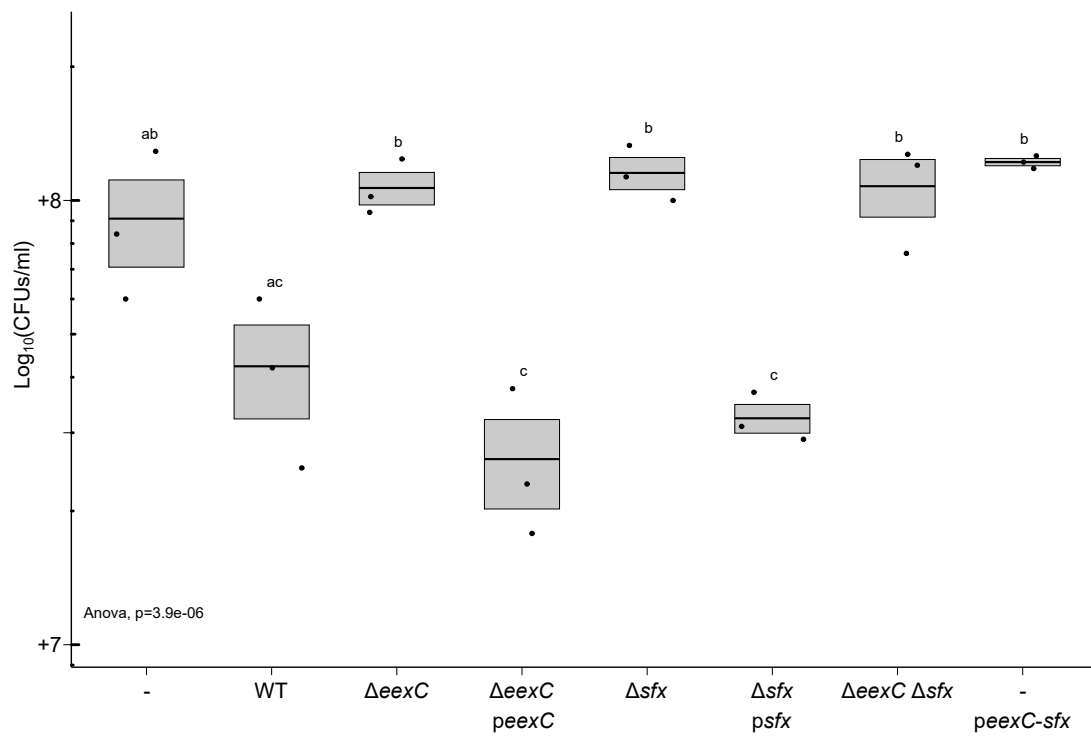

Supplement: S2 Fig — Colony forming units (CFUs) were obtained from mating assays conducted in Fig 1D. Crossbars show the mean and standard error of the mean of three independent experiments. One-way ANOVA (p = 3.9e-6) with a Tukey-Kramer post-test was used on the log10-transformed values to compare the means. Statistical significance (S2 File) is shown as a compact letter display for pairwise comparisons where means grouped under identical letters are not statistically different. (PDF) [file pgen.1011442.s002.pdf]

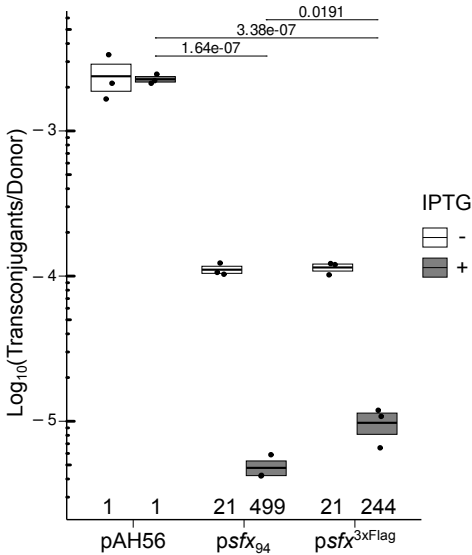

Supplement: S4 Fig — pVCR94Sp was transferred from E. coli VB111 (Nxr) into E. coli DH5α λpir bearing the indicated pAH56 derivatives (Knr) expressing sfx94 or sfx94-3xFlag in the presence or absence of IPTG. Transconjugants containing pVCR94Sp were selected as the Knr Spr colonies. Crossbars show the mean and standard error of the mean of three independent experiments. One-way ANOVA (p = 7.3e-8) with a Tukey-Kramer post-test was used on the log10-transformed values to compare the means for the IPTG-induced condition. (PDF) [file pgen.1011442.s004.pdf]

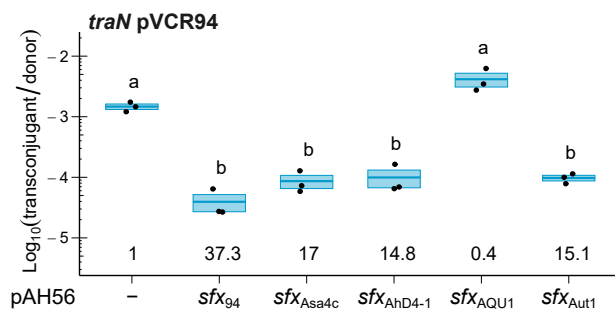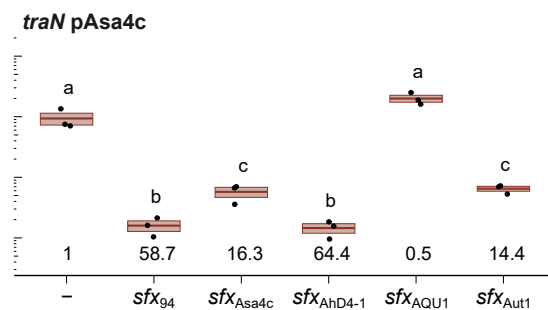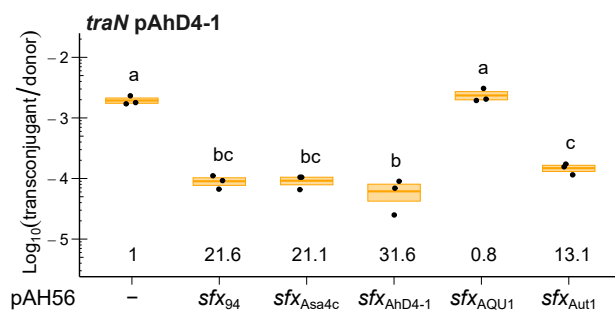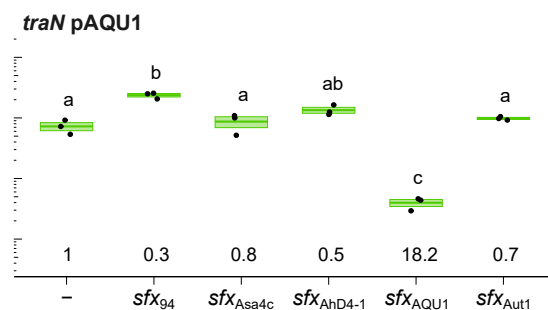

Supplement: S5 Fig — E. coli GG56 (Nxr) containing pVCR94Sp ΔtraN and expressing the indicated traN variants from pBAD30 (Apr) served as the donor, and E. coli CAG18439 (Tcr) expressing the indicated sfx variants from a single-copy, chromosomally integrated pAH56 (Knr) was used as the recipient. Transconjugants containing pVCR94Sp ΔtraN were selected as the Tcr Spr colonies. Crossbars show the mean and standard error of the mean of three independent experiments. One-way ANOVA (traN pVCR94, p = 2.21e-8; traN pAsa4c, p = 4.84e-10; traN pAhD4-1, p = 2.20e-8; traN pAQU1, p = 1.81e-9) with a Tukey-Kramer post-test was used on the log10-transformed values to compare the means. Exclusion indices, shown at the bottom of each crossbar, are calculated by dividing the frequency of transfer of pVCR94Sp ΔtraN to the empty recipient divided by the transfer frequency to the recipient expressing the indicated sfx variant. Statistical significance (S2 File) is shown as a compact letter display for pairwise comparisons where means grouped under identical letters are not statistically different. (PDF) [file pgen.1011442.s005.pdf]

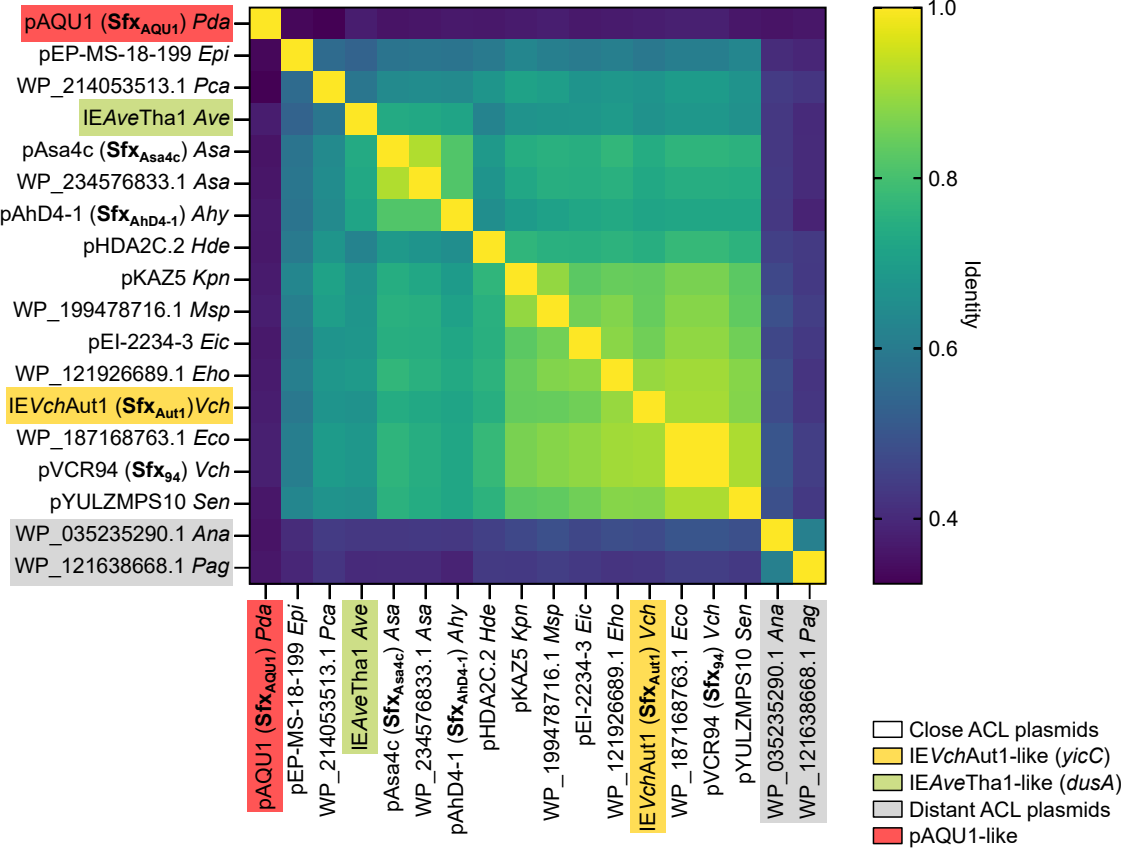

Supplement: S6 Fig — Panels are shaded as indicated in the figure according to the identity calculated for each pair. (PDF) [file pgen.1011442.s006.pdf]
