# Supplementary material for: Surface exclusion of IncC conjugative plasmids and their relatives
Source: PLoS Genet. 2024 Oct 9;20(10):e1011442. doi: 10.1371/journal.pgen.1011442 (PMC11493245; doi:10.1371/journal.pgen.1011442)
Supplement: S3 Fig — (A) Schematic representation of the predicted translation products of sfx94 and traN94 and their predicted signal peptide sequences. (B) Predicted Rho-independent terminators in the proximity of sfx in the IncC plasmid pVCR94. Terminator sequences are provided with the stems and loops in blue and red, respectively. Their position is represented by hairpins on the schematic representation of the region. Predicted ΔG values are indicated in kcal/mol. (PDF) [file pgen.1011442.s003.pdf]

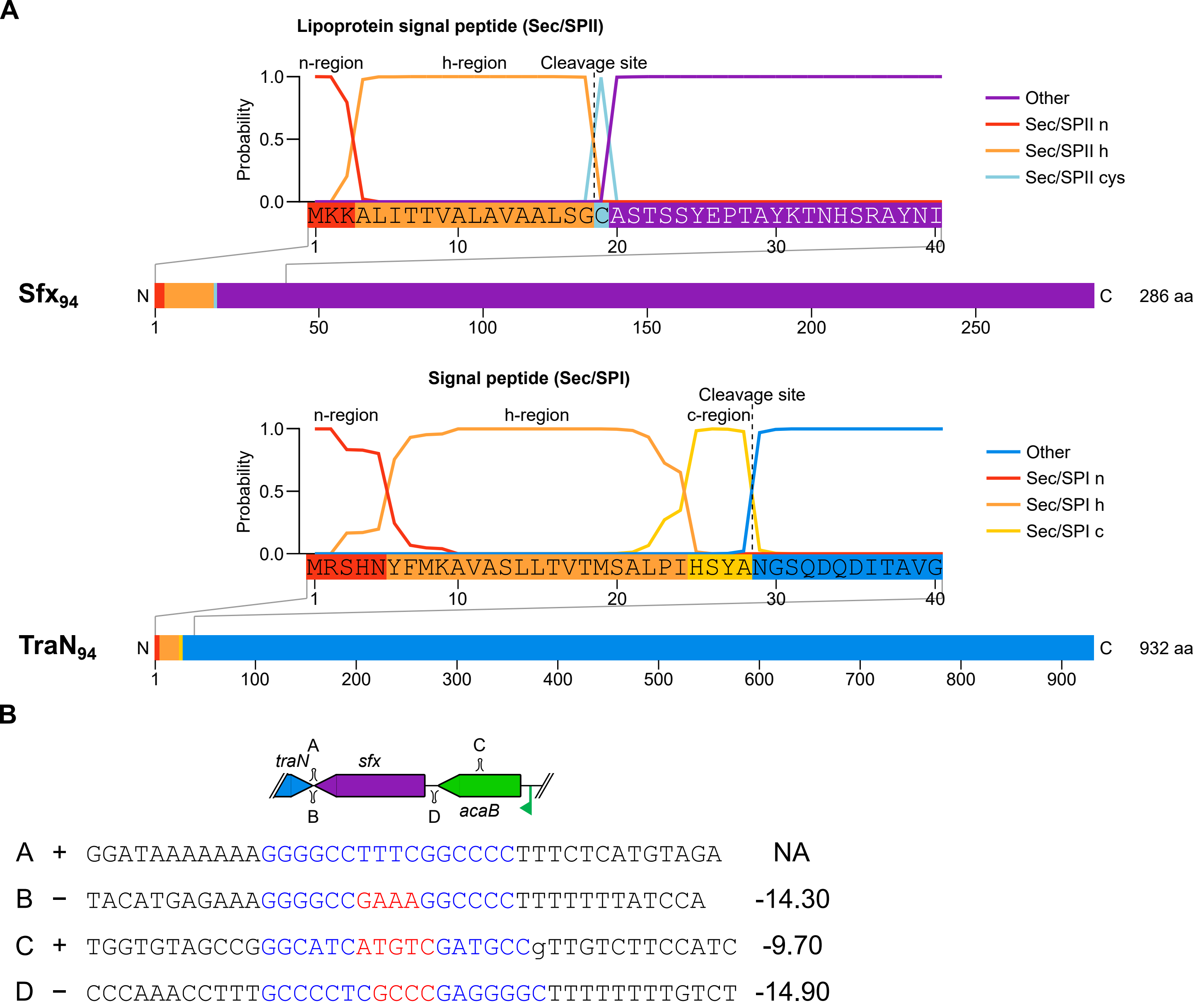
A
Lipoprotein signal peptide (Sec/SPII)
n-region
h-region
Cleavage site
Probability
1.0
0.5
0.0
MKKALITTVALAVAALSGCASTSSYEPTAYKTNHSRAYNI
1
10
20
30
40
Other
Sec/SPII n
Sec/SPII h
Sec/SPII cys
Sfx94
N
C
286 aa
1
50
100
150
200
250
Signal peptide (Sec/SPI)
n-region
h-region
Cleavage site
c-region
MRSHNYFMKAVASLLTVTMSALPIHSYANGSQDQDITAVG
Other
Sec/SPI n
Sec/SPI h
Sec/SPI c
TraN94
932 aa
100
200
300
400
500
600
700
800
900
B
traN
sfx
acaB
A
B
C
D
A + GGATAAAAAAAGGGGCCTTTCGGCCCCTTTCTCATGTAGA NA
B − TACATGAGAAAGGGGCCGAAAGGCCCCTTTTTTTATCCA -14.30
C + TGGTGTAGCCGGGCATCATGTCGATGCCgTTGTCTTCCATC -9.70
D − CCCAAACCTTTGCCCCTCGCCCGAGGGGCTTTTTTTTGTCT -14.90
