# Supplementary material for: Surface exclusion of IncC conjugative plasmids and their relatives
Source: PLoS Genet. 2024 Oct 9;20(10):e1011442. doi: 10.1371/journal.pgen.1011442 (PMC11493245; doi:10.1371/journal.pgen.1011442)
Supplement: Attachment — Submitted filename: Response to reviewers.docx [file pgen.1011442.s012.docx]

**Reviewer #1:** Many conjugative plasmids are known to have mechanisms that exclude acquisition of another copy of the plasmid, or other DNA that might be transferred through the plasmid encoded secretion system. This manuscript describes the identification of a previously unknown exclusion gene found on many IncC and IncA-type plasmids. I believe that there are three main findings in this manuscript.

1. The identification of a plasmid gene, named sfx, that confers exclusion (presumably surface exclusion) for IncC-like plasmids in Gamma proteobacteria. A TraDIS/Tn-seq approach was used to identify exclusion genes in the absence of the known exclusion gene eexC. Sfx appears to be an outer membrane lipoprotein that is constitutively expressed.

I had a difficult time understanding how the insertion library was made. Were insertions only in the plasmid? Or, were insertions in the plasmid and throughout the genome? In the first case, I would presume that insertions were made in a plasmid-containing strain, generating mini-Tn5 insertions in the genome and in the plasmid. Then the plasmid was transferred to an unmutagenized strain, selecting for the mini-Tn5. In this way, mutations in the 'library' were limited to the plasmid. Or, the original strain with insertions was used as the library, in which case there were mutations in both the chromosome and the plasmid. In either case, this should be clarified with a simple summary in the results and more detailed description in the methods. If the insertion library contained insertions in both the plasmid and the chromosome, then additional analyses and discussion of host genes identified is important to include. Specifically, were there host genes identified that influenced the efficiency of transconjugant formation?

We apologize for the lack of details as we thought the cartoon in Fig 1A and the description in the Materials and methods made the TraDIS approach easily understandable. The input library used as the recipient in the TraDIS experiment contains Tn*5* insertions in both the plasmid and the chromosome. This is now made clearer in the result section (lines 145-147 and 151-152). We emphasize that we do not monitor the movement of the IncC plasmid between the donor strain and Tn*5*^+^ recipient library due to plasmid incompatibility. We only followed the entry of the mobilizable plasmid pClo. For this reason, we only looked for Tn*5* insertions in pVCR94^Kn^ Δ*eexC* that enhanced the mobilization of pClo. The involvement of chromosomal genes in the transmission of IncC plasmids is an interesting topic that will be addressed in future works and is beyond the scope of this manuscript.

Is sfx the only plasmid gene needed for exclusion? In other words, does expression of sfx in the absence of the plasmid, confer exclusion? Same for eexC? this might be in Fig4B, but from the description of the experiment in the results, and the figure legend, I could not tell which strains were used as donors and recipients, nor whether there recipients contained a conjugative plasmid. I think the recipients did not contain a plasmid and expressed sfx alone, but this should be made clear and explicitly. Further, if this is the case, then it means that sfx is sufficient to confer exclusion, a point worth making.

Yes, *sfx* or *eexC* without the IncC plasmid contribute each to exclusion and no other gene is required. To strengthen our conclusion, we cloned *sfx* and *eexC* in the single copy vector pBeloBAC11 under the control of their respective native promoters. As shown in the new Fig 1D, the level of exclusion exerted by pBeloBAC11 bearing *sfx* and *eexC* is identical to the level obtained with pVCR94.

2. sfx homologues were found in IncA and IncC plasmids, and interestingly, in mobilizable elements. Although it is not known if any of the homologues in the mobilizable elements are functional, this finding is intriguing. As discussed in the manuscript, this could have implications for the transfer and spread of these mobilizable elements, and their impact on the spread of conjugative plasmids.

We improved on this by testing the *sfx* gene found on IE*Vch*Aut1 and showing that it excludes conjugative plasmids encoding *traN* of the IncC group, but not plasmids such as pAQU1 (Fig 4C and S5 Fig). Hence *sfx* of these mobilizable elements is functional.

3. The plasmid-encoded TraN (a putative adhesin) determines the specificity of Sfx-mediated exclusion. The experiments supporting this are clear. However, there are contradictory statements in several parts of the manuscript as to the interpretation. In one interpretation, stated in the abstract, "the putative adhesin TraN in the donor is the target of surface exclusion". This implies direct interaction. In contrast, there is a statement in the discussion that "direct contact between Sfx and TraN is unlikely for surface exclusion." The statement that TraN determines the specificity of Sfx-mediated exclusion seems accurate, at least for the examples tested, and this specificity could be due to direct or indirect interactions. Indirect would be Sfx 'shielding' a host-encoded surface receptor with which TraN interacts, and, TraN from different plasmids would necessarily interact with different surface receptors. There should be consistent descriptions/inferences throughout the manuscript.

We have modified the abstract and carefully edited the text to make it clear that Sfx and TraN pairs are the players determining surface exclusion specificity without inferring direct interactions between the partners.

Regarding expression of sfx and eexC; Expression is measured by determining the amounts of mRNA for each gene. This might or might not be reflective of relative protein accumulation. Are there data for relative protein amounts? Oligomeric state of each protein? I'm not suggesting that these experiments be done unless easy to do. Rather, I think that it is a leap to go from different levels of experssion to one target (EexC) being on the donor and Sfx acting to shield a receptor on the recipient.

We did not quantify the amount of mRNA, but translation levels. There were inconsistent descriptions of the constructs in the text and legend of Fig 2 that misled the reviewers. We apologize for these inconsistencies. Unlike stated in the legend, the expression of *sfx* and *eexC* was measured using translational fusions to *lacZ*, not transcriptional fusions. We have now corrected the legend and provided additional information on the constructs in the methods (translational fusion of the 3^rd^ codon of *sfx* and *eexC* to the 8^th^ codon of *lacZ*) in lines 479-480 and Table 2. Since we directly measured the expression of the translational fusion of *sfx* and *eexC* from the native promoters on pVCR94, our comparison considers both the amounts of mRNA and translational regulation by the RBS.

Further, how much TraN is produced in donors? It might be much more abundant than anticipated.

We added a translational *traN*’-*‘lacZ* fusion (Fig 2D) to monitor the expression of *traN* and found that its expression is barely detectable in IncC+ cells in pure culture, that is at a much lower level than *sfx*.

The conclusion that Sfx is causing surface exclusion is not based on a direct experiment measuring surface exclusion. I'm not suggesting such an experiment. However, I think an explicit statement as to why it is likely surface exclusion is needed. It is inferred to be surface exclusion because specificity is determined by TraN, a putative adhesin, and (I believe) to a much lesser extent, that Sfx is on the cell surface.

We made clearer in the discussion that surface exclusion was inferred from the cell fractionation assays which localize Sfx in the outer membrane and the exclusion specificity was determined by *sfx*/*traN* pairs (lines 352-356).

Are there measurable consequences to cells that contain the plasmids missing sfx, eexC, or both? Are these systems beneficial for the plasmid alone, or the cells too?

We added a new S2 Fig, showing that no detectable negative impact of the absence of *eexC*, *sfx* or both on the CFU counts in our experimental setting. This does not mean that there is no consequence in a more natural context. Fig 2D shows that the expression of *traN* is nearly undetectable in a pure culture of IncC+ cells, confirming a very tight control of conjugative transfer (alleviated by *acaCD* expression), that likely prevents spurious transfer of IncC plasmids between donors and prevent us from detecting adverse effects of abolishing entry and surface exclusion. This is now stated in the discussion (lines 440-444).

Other comments

line 31, 35 (and elsewhere?); Perhaps avoid 'novel'. It is sufficient to say unrelated to ones described to date...

We edited the text accordingly and all instances of “novel” protein or family have been removed.

l 120/ 757; There are inconsistencies throughout in usage of Tn-seq (l 405, 426, 757) versus TraDIS(l 120, 151, 398, 514, 811) – likely other places. Conceptually, these approaches are similar, but my understanding is that there are some technical differences. Please be consistent throughout with the description.

We have modified the text to consistently use TraDIS throughout the manuscript.

l 135-136; Tn5 library; Normal Tn5 encodes kanamycin resistance and the plasmid used is KanR. Please indicate that a mini-tn5 spc was used. Indicate spectinomycin resistance in the description in results.

We have now indicated that mini-Tn5 is conferring spectinomycin resistance in line 143.

As noted above, construction of library of mini-tn5 insertions was not at all clear to me. was library made in recipient that had pVCR94kan? or library made in cells with pVCR94kan and then pVCR94kan with mini-Tn5(spc) mated into a clean strain selecting for mini-tn5 spc? The cartoon in Fig1A did not clarify this, and if I interpret the cartoon at face value, it appears that the insertion library was made in recipients that contain the plasmid and would contain insertions in both the plasmid and the chromosome. This might be true, but should be stated explicitly.

As indicated above, we have modified the text to make it clear that the input library containing insertions in both the plasmid and the chromosome was used as the recipient, as shown in Fig 1A, which was correctly interpreted by the reviewer.

l 143-150; Table 1; regarding the nine candidates from insertion library, but deletion of only one of the nine genes caused a phenotype: It might be useful to include some brief ideas/speculation as to why others identified in library were not real. Perhasp there were multiple insertions in those strains? Other mutations?

Small gene size and relatively low saturation of the input library increased the likelihood of spurious insertions in the output library coming out as significant hits.

All figures. Statistics on graphs unclear (are matching letters statistically significant or not significant?). Please clarify statistics in figure legends. Also, it seems that pairwise comparisons might be more useful than the Anova analysis?

We now describe better the use of the compact letter display for the statistical significance of the comparisons. Anova is the way to go in most of our analyses (multiple comparisons between groups). Pairwise comparison is only valid in the case of Fig 4B.

For figures with mating experiments; please make clear donor and recipient strains and relevant properties. The figure legend should include the strain numbers (and relevant properties) so a reader can figure out what used for each experiment.

All legends of figures with mating experiments have been carefully edited to include all relevant properties and description of donor and recipient strains.

Fig2F. The colors used are very difficult to distinguish from each other (at least for me). If possible, use colors with much greater contrast.

We increased the contrast of the color differences in the figures where necessary.

There is a lot of nomenclature/jargon that will be difficult for many to understand. Some more general description of the terms would be helpful. For example: SGI1 (salmonella genomic island 1); paragraph starting on l 194; should briefly describe MGIBchHai6 family and dusA; what is relevance/significance of these?

We have modified the text accordingly in the introduction to be more descriptive of SGI1 and MGI*Vch*Hai6 (lines 68-71). Descriptions of *dusA*, *yicC* and *rlmD* have been introduced in the corresponding result section (lines 221, 224 and 229).

l 213/ fig 4. “modest yet significant decrease was observed for the ΔeexC Δsfx mutant”. Is this also not true for the Δsfx mutant alone?

We modified the text accordingly, now in line 292.

Fig4; C. The exclusion index should be briefly described in the legend.

Exclusion indices are now described in the legend of Fig 4.

l 237; "All conjugative plasmids encode at least on exclusion factor [12]"; I suggest qualifying this statement. It is impossible to know 'all'.

We only conveyed the conclusion of the review by Garcillán-Barcia and de la Cruz. We changed the statement to “All known conjugative plasmids” (line 339).

l 800. “The presence of eexC and sfx in the donor decreases the frequency of transconjugant formation”. Based on the data shown, this should be increases (not decreases; WT has more transconjugants than eexC and sfx mutants).

The reviewer is correct, we meant “The absence of *eexC* and *sfx*”. Our description in the legend was incorrect but the interpretation in the result section was correct. We modified the legend of Fig 4A accordingly.

Fig S1; There appears to be a decrease in insertions in traB in the transconjugant (output) population. If so, does this imply that the presence of traB in recipients stimulates conjugation?

The apparent decrease of *traB* insertions in the output seemingly results from a non-reproducible enrichment of insertions in the input library (visible in the first but not in the second replicate). Investigations are ongoing.

**Reviewer #2:** The exclusion phenomenon prevents the de novo acquisition by conjugation of plasmids that are identical (or related) to other plasmids already present in the recipient cell. This mechanism is critical to the dissemination dynamics of conjugative elements in bacterial populations.

IncC plasmids, like most (if not all) conjugative plasmids, encode an entry exclusion protein Eex. However, surface exclusion proteins are less frequently reported on conjugative elements. In the manuscript "Surface exclusion of IncC conjugative plasmids and their relatives", Rivard and co-authors use an elegant high-density transposon-directed insertion sequencing screen to identify a novel surface exclusion factor of IncC plasmids, Sfx. Sfx is responsible for the exclusion activity not attributable to the Eex factor. Bioinformatic analysis shows that the identified Sfx gene is unrelated to those already described for other conjugative plasmids. Notably, the authors demonstrate that homologues of this new type sfx gene are found in a range of Gammaproteobacteria plasmids belonging to IncC and IncA plasmids, but also in untyped conjugative plasmids and a novel family of IME (integrative and mobilizable elements). Importantly, the work also provides evidence that the putative adhesin TraN produced in the donor cell and exposed at the cell surface is the (direct or indirect) target of surface exclusion by Sfx exposed at the surface of the recipient cells.

The manuscript is well-written and logical. The experimental strategy and the results are well presented and accompanied by precise figures. However, this reviewer would like to raise one concern.

Throughout the manuscript, the authors present the conjugation efficiency as Log10(transconjugants/donors), from which they interpret the exclusion activity of the different constructs/genes tested. However, exclusion activity is usually estimated by calculating the exclusion index, which corresponds to the ratio of an element's transfer frequency toward an empty recipient to its transfer frequency toward the tested recipient.

Therefore, this reviewer recommends calculating and presenting these exclusion indexes in place of, or in addition to Figures 1D, 4A and 4B (this might also apply to Fig. S4). Alternatively, the authors should explain why they have decided not to calculate and show these estimates for the corresponding experiments.

We thank this reviewer for his remark. We used exclusion indices in the heatmap of Fig 4C, but now realize that it makes sense to use it more broadly. However, to retain details regarding transfer frequencies that would be lost if we replaced these figures with exclusion indices only (a ratio of frequencies), we decided to keep the figures expressing the transfer frequencies and to indicate the numerical value of the exclusion index for each strain where appropriate.

This presentation choice is even more surprising that the authors have calculated the exclusion index for the experiments addressing sfx/traN genetic interaction presented in the checkerboard Figure 4C. This figure's exclusion index values appear very low (scale from 0-white to 1-brown). This is surprising since exclusion indexes associated with exclusion factors are usually in the range of 10, 100 or even 1000. If this reviewer is correct, Figure 1D indicates that the exclusion index associated with eex and sfx genes would be somewhere around ~500, as calculated from (Conjugation efficiency towards the strain without plasmid (approx. 10-2)) divided by (Conjugation efficiency towards the strain containing pVCR94Spwt (almost. 10-5)). The authors should explain why the exclusion index shown in Figure 4C are so low. Are these normalized? How are they calculated?

We agree with this reviewer. We now provide all exclusion indices for all but Fig 4A. Exclusion indices are calculated by dividing the frequency of transfer of a plasmid into an empty recipient strain by the frequency of transfer of the same plasmid into a recipient strain of interest. For Fig 4C, we forgot to indicate in the legend that the exclusion index scale was a log10 transformation. We corrected the figure using a linear scale and provided the exclusion index values directly in the heatmap.

Difference of exclusion index values for pVCR94 between Fig 1D and Fig 4C (and S5 Fig) can be attributed to the difference of expression of *traN* and *sfx* from their native promoters in Fig 1D and from the arabinose-inducible promoter for *traN* in donors and IPTG-inducible promoter for *sfx* in recipients in Fig 4C (and S5 Fig).

The presentation of exclusion indexes in this work is critical since variation in the Log10(transconjugants/donors) values can be attributed to variation in conjugation rate indeed but also accounts for variation in the viability/fitness of donor, recipient and transconjugant cells in the mating culture. This brings about the second major comment.

The authors do not mention the physiological effect of exclusion systems inactivation on the strain's fitness/growth/viability. Without doing additional experiments, these effects should be revealed by (buried in) the number of colonies (Cfu/ml) obtained during the plaiting of conjugation mixes on selective media, from which Figure 1D, Figure 4A, B and C have been generated. For instance, if we focus on Figure 4A, similar Log10(transconjugants/donors) values are obtained for pVCR94Sp WT and eexC-, which does not mean that the absolute number of colonies per plate is identical (and might or might not be different). Indeed, this reviewer would expect the deletion of exclusion genes to be associated with a viability defect of donor and transconjugant cells due to unregulated self-transfer. Did the authors observe any viability/growth/fitness defect related to the deletion of eex and/or sfx genes? Whether or not it is the case, the authors should consider presenting viability estimates and commenting on this crucial point.

We did not observe any negative impact related to the removal of surface and/or entry exclusion on the viability or fitness of donor cells, unlike was reported for the F plasmid. S1 File (Tab Fig 1D) and the new figure S2 Fig show no statistically significant difference between CFU/ml for WT, Δ*eex*, Δ*sfx*, and Δ*eex* Δ*sfx* cells. Conversely, overexpression of *sfx* or *eexC*, negatively impacted the CFU counts. This is now presented in the results (lines 190-191) and discussion (lines 347-351).

Overall, this work constitutes an important addition to the field of conjugation biology. Not only do the authors uncover a new type of surface exclusion protein, but they also demonstrate its conservation in a range of conjugative elements and provides convincing insights into its mechanism of action. The observed phenotypic interaction between the surface exclusion factors Sfx and TraN is novel and reveals the diversity of exclusion mechanisms amongst conjugative elements.

Minor comments:

-	Figure 1D. The authors should consider presenting the results for

the double complementation of pVCR94 deleted for eex and sfx by pAH56 carrying both eex, sfx.

As requested by the reviewer, we carried out the double complementation of *eex* and *sfx* from their native promoter on pBeloBAC11, restoring full exclusion.

-	Figure 4A and B. The authors should indicate in the figure legends

what the letters (a, b, ab in panel A and bc, a, b, an c in panel B) correspond to.

We have edited the legend of these figures to indicate that statistical significance is shown using the compact letter display (CLD) method so that means with the same letter are not significantly different from one another.

-	Figure 4C. Why are the index values so low?

We apologize for the confusion. We forgot to indicate in the legend that the heatmap corresponds to range calculated as the log10 of exclusion index. We now provide an updated heatmap with an additional Sfx representative, a linear scale, and the exclusion index values for each tested *traN*/*sfx* pair.

**Reviewer #3:** The Rivard, Humbert, et al manuscript is interesting and outlines some important observations, including the description of the Sfx family of conjugative exclusion factors and identification of its target in a recipient cell. The TraDIS assay clearly shows the importance of Sfx and EexC to antagonize conjugation between IncA/C-containing bacterial donors. Further work with deletion and complementation studies show that Sfx carries out surface exclusion via specific interactions with a cognate outer membrane adhesin TraN. Overall, the authors do a good job with the background and significance, and place their results/discussion in a clear context for the field.

We thank the reviewer for his comments.

In general, the Figures & Figure legends need some careful editing and revision: I could not decipher the meaning of the labels "a, b, c" in Fig1D or in Fig 4A/B.

We have edited the legend of these figures to indicate that statistical significance is shown using the compact letter display (CLD) method so that means with the same letter are not significantly different from one another. Details regarding p values are shown in S2 File.

The labeling of Fig 4A vs 4B was quite confusing--in 4A the x axis labeling refers only to genes in the recipient, while in 4B, the x axis has both donor and recipient genes indicated. It isn't that complicated, but it took several readings for me to sort it out. My understanding of the narrative on Fig 4A was further complicated by the contradictory statements in lines 212-213 vs 799-800--as I understand it, lines 799-800 should be corrected to say "(A) The ABSENCE of eexC and sfx in the donor decreases the frequency of transconjugant formation." --not the presence.

Indeed, we incorrectly stated “presence” instead of “absence” in the legend. We corrected the legend accordingly. The interpretation in the results section was however correct and remains unchanged. To make the panels A and B easier to understand, we improved the labeling and now indicate that the pVCR94Sp mutants were the donors (D) in Fig 4A and that *traN* was expressed in donors (D) and *sfx* was expressed in recipients (R) in Fig 4B.

Fig 2 needs work: in Fig. 2A, the proposed cellular localization of Sfx is probably correct, but the data shown in Fig. 2A is inadequate. Without more information on the cellular fractionation and how where other proteins with known localization are behaving in this experiment, the statement in lines 245-246 isn't really conclusively shown. The outer membrane lane in the stained portion of the gel is uninterpretable; in the Western blot portion, you should show that there is more Sfx in the outer membrane fraction than in other cell fractions--showing a "total protein" and OM sample is insufficient.

We changed our approach for the localization of Sfx. We now provide a new Fig 2A, showing that Sfx localizes in the OM (like OmpC), but it also accumulates in the inner membrane in our setup, likely due to ectopic overexpression of 3xFlag-labelled Sfx. OmpC and AcaC serve as the controls for the outer membrane and cytoplasmic fraction, respectively. The complete western blot and silver-stained gel from which Fig 2A was built are provided for review.

The expression of acaB (2D) from a different cDNA could be added to verify that the acaB gene is expressed from its expected promoter by AcaCD.

We did not carry out the requested assay as the regulation of *acaB* as part of the AcaCD regulon has been characterized extensively in previous papers by our group and others (PMID 33834206, 25340549, 32807890).

I'm not sure that Fig 2E adds anything beyond Fig 2F.

Fig 2E (β-galactosidase activity on LB agar) was removed.

Overall, it seems likely that Sfx is more highly expressed than EexC, but the transcriptional LacZ data would miss any translational control signals--given the uncertainty of the promoter for sfx, it seems prudent to acknowledge the limitations of this data (see lines 326-327).

As stated in the remark made by reviewer 1, there were inconsistent descriptions of the constructs in the main text and legend of Figure 2 that misled the reviewers. We apologize for this. Unlike stated in the legend, the expression of *sfx* and *eexC* was measured by translational fusion to *lacZ*, not transcriptional fusion. We have now edited the manuscript accordingly. Since we directly measured the expression of the translational fusion of *sfx* and *eexC* from the native promoters on pVCR94, both transcriptional and translation control signals are considered in the reported expression. Furthermore, we have added a translational *lacZ* fusion to *traN* for comparison of the level of expression with an AcaCD-responsive gene.
